# Detecting social cues conveyed by laughter and associations with callous-unemotional traits in early childhood

**DOI:** 10.1016/j.jecp.2025.106395

**Published:** 2025-10-16

**Authors:** R.C. Plate, M. Flum, Y. Paz, E.R. Perkins, Y. Rodriguez, J. Herrington, J. Parish-Morris, R. Waller

**Affiliations:** a Department of Psychology, University of Pennsylvania, Philadelphia, PA, USA; b Center for Autism Research, Children’s Hospital of Philadelphia, Philadelphia, PA, USA; c Department of Child and Adolescent Psychiatry and Behavioral Sciences, Children’s Hospital of Philadelphia, Philadelphia, PA, USA; d Department of Psychiatry, Perelman School of Medicine, University of Pennsylvania, Philadelphia, PA, USA

**Keywords:** Laughter, Affiliation, Dominance, Emotion, Development

## Abstract

Beginning in infancy, laughter promotes positive social interactions. However, laughter can also convey derision. Adults distinguish friendly from derisory laughter, appropriately modulating social behavior based on their perceptions of the laugher’s intent. Given the connection between laughter perception and broader social functioning, it is important to understand how this skill develops in young children who are initiating foundational early social bonds. Moreover, children who have difficulties forming and maintaining social bonds—including those with callous-unemotional (CU) traits—may show differences in correctly identifying and responding to laughter. Here, 3-, 4-, and 5-year-old children (*N* = 150; 50% female) categorized laughter clips that varied in conveying affiliation (i.e., friendly) or dominance (i.e., mean) and reported on whether they wanted to play with the person producing the laughter. Children distinguished between laughter types, were accurate at detecting mean laughter, and showed increasing accuracy across ages 3 to 5 years. Children expressed a preference to play with friendly versus mean laughers, a distinction that sharpened from ages 3 to 5 years. Higher CU traits predicted lower accuracy for identifying mean laughs, with no CU-related difference for friendly laughs, though CU traits were not related to social preference. The findings provide the first evidence of young children’s ability to detect and appropriately adjust their behavioral intentions based on different communicative signals conveyed in laughter. Findings also suggest that these abilities may be relevant to young children who have difficulties with interpersonal interactions and social bonding.

## Introduction

Laughter is a universal social signal (Addyman et al., 2018; Addyman & Addyman, 2013; Chapman, 1973; Martin, 2002; Provine, 1993; Provine, 2001) that frequently occurs during interpersonal interactions (Stieger et al., 2024). Laughter promotes social bonding and psychological well-being by connecting social partners experiencing the same positive emotional states (Dunbar et al., 2021; Dunbar & Mehu, 2008; Dunbar et al., 2012; Cekaite & Andrén, 2019). However, laughter can also indicate negative social feedback when its function is teasing, taunting, or mocking (Provine, 2001; Winkler & Bryant, 2021). Two well-studied social functions of laughter are to convey *affiliation* (i.e., connectedness, friendliness) and *dominance* (i.e., control, meanness). Each type of laughter is associated with unique acoustic properties and communicates specific messages to social partners; adults distinguish between these types of laughter (Plate et al., 2022; Wood et al., 2017; Wood & Niedenthal, 2018). However, no studies have investigated individual differences in young children’s ability to detect and respond contingently to differential social signals conveyed by laughter.

The importance of laughter for social development begins in infancy (Addyman & Addyman, 2013). Laughter contributes to positive social interactions between infants and caregivers (Vouloumanos & Bryant, 2019; Winkler & Bryant, 2021), and infants listen longer to colaughter between friends than between strangers engaging in a positive social interaction (Vouloumanos & Bryant, 2019). Further, infants who better differentiated between sounds of crying versus laughter had better emotion regulation (Crespo-Llado et al., 2018). Thus, infants appear to have expectations about how the vocal signals conveyed by laughter map onto social behavior, and early individual differences in responsivity to or expression of laughter appear to relate meaningfully to variations in temperament and behavioral intentionality relevant to social functioning.

In early childhood, laughter is often observed during social play, serving an important function for developing social bonds (Addyman et al., 2018; Davila-Ross & Palagi, 2022; Leavens, 2009). For example, 2–4-year-old children laughed more when watching a funny video in the presence of other children than while alone (Addyman et al., 2018). Importantly, children use their ability to recognize social signals to make predictions about the behavior of others (Plate et al., 2022; Ruba & Pollak, 2020; Woodard et al., 2022) and adapt their own behavior accordingly. For example, between ages 2–5 years, children improve in their ability to distinguish genuine from fake smiles and 4–5-year-olds infer that people displaying genuine smiles will act more prosocially than those displaying fake smiles (Song et al., 2016). By age 5, children become more discerning and strategic in directing prosocial behavior to specific individuals to benefit their own goals (Grueneisen & Warneken, 2022). In conjunction with other social cues, laughter appears to be fundamental to our ability to build and maintain complex interpersonal relationships, including as a social cue that children use to guide their behaviors (Winkler & Bryant, 2021).

Laughter is also a fruitful target for understanding difficulties in socioemotional functioning. For example, adults high on psychopathic traits (i.e., manipulative, callous, impulsive) showed poorer recognition accuracy when distinguishing between different laughter cues, while reporting wanting to join in more with laughter, particularly mean laughter (Plate et al., 2022). In contrast, some children and adolescents show extreme fear of being *laughed at* (“gelotophobia”) (Proyer et al., 2012; Ruch & Proyer, 2009), Gelotophobia is associated with being bullied or victimized (Proyer et al., 2013) and occurs at higher rates among children with high-functioning Autism Spectrum Disorder (ASD) (Leader et al., 2018; Samson et al., 2011). Gelotophobia is also associated with a heightened tendency to misinterpret laughter as having a negative intent (Kreifelts et al., 2014; Ruch & Proyer, 2009), while children with higher empathy are better at distinguishing authenticity in laughter intent (Neves et al., 2018). Together, these studies suggest that variability in responses to laughter may provide clues to mechanisms underlying difficulties in interpersonal interactions across different forms of psychopathology, including in children.

Callous-unemotional (CU) traits (i.e., low empathy and guilt) are relevant to children’s ability to detect social intentionality in laughter, because laughter conveys both positive behavioral intention (e.g., encourages affiliation and reciprocal positive affect) and negative behavioral intentions (e.g., assert dominance or mocking, convey negative affect; Wood et al., 2017; Wood & Neidenthal, 2018). Children with CU traits are characterized by interpersonal difficulties (Matlasz et al., 2020) and are at high risk for later violence and crime (Cardinale & Marsh, 2020; Frick et al., 2014; McMahon et al., 2010). CU traits are often described as representing a developmental precursor to the affective features of psychopathy (i.e., uncaring, shallow affect) and have been broadly linked to emotion recognition difficulties for cues of fear or sadness, including from faces (Powell et al., 2023; Bedford et al., 2017; Marsh & Blair, 2008; Moore et al., 2019), body postures (Muñoz, 2009), and vocal expressions (Dawel et al., 2012).

However, despite evidence linking difficulties with laughter recognition to psychopathic traits in adults (Plate et al., 2022; Torres-Marín et al., 2019), we lack studies that have investigated how CU traits relate to the recognition and response to laughter in children. In one exception, boys aged 11–16 years old with disruptive behavior disorder and CU traits were as good as typically developing peers at distinguishing genuine versus posed laughter, but they reported less interest than peers in joining in with genuine laughter (O’Nions et al., 2017). Difficulties recognizing and responding appropriately to laughter may explain some of the interpersonal difficulties associated with CU traits even in early childhood, especially since such difficulties may relate to core characteristics associated with CU traits, including reduced emotional reactivity (Truedsson et al., 2019), lower empathy (Waller et al., 2020), more proactive aggression (Marsee & Frick, 2010), and difficulties establishing social bonds (Miron et al., 2020; Waller & Wagner, 2019).

To understand when the ability to distinguish different communicative intentions of laughter emerges, we adapted a laughter categorization task (Plate et al., 2022) for 3- to 5-year-old children. In a within-subjects design, children categorized laughter varying in the degree of affiliation and dominance as friendly or mean. Children also indicated whether they wanted to play with each laugher (i.e., social preference). In our first aim, we assessed recognition accuracy and social preference, hypothesizing that children would accurately recognize friendly and mean laughter above chance and express greater social preference for friendly laughers, with higher accuracy and stronger social preferences with increasing age. In our second aim, we investigated whether CU traits relate to laughter recognition accuracy and social preference, hypothesizing that children higher in CU traits would show poorer discernment between friendly and mean laughter and reduced social preference for friendly laughers.

## Method

### Participants

The sample included 150 participants (age 3, *n* = 54, 56 % female; age 4, *n* = 48, 50 % female; age 5, *n* = 48, 44 % female) drawn from an online study, which recruited 162 children with one of their parents, primarily from a large city in the Northeastern United States.^1^ Of the original 162 participants, 1 did not begin the task, 7 were excluded for technical issues, 3 exhibited a fixed response style in the recognition task, and 1 was missing the entire CU traits measure. Sample size was determined by a power analysis for a similar task that children completed during their visit (Paz et al., 2025), and the age range was selected based on prior research showing that children undergo rapid prosocial development from ages 3–5 years (Grueneisen & Warneken, 2022).

### Measures

#### Laughter task

**Fig. 1.**
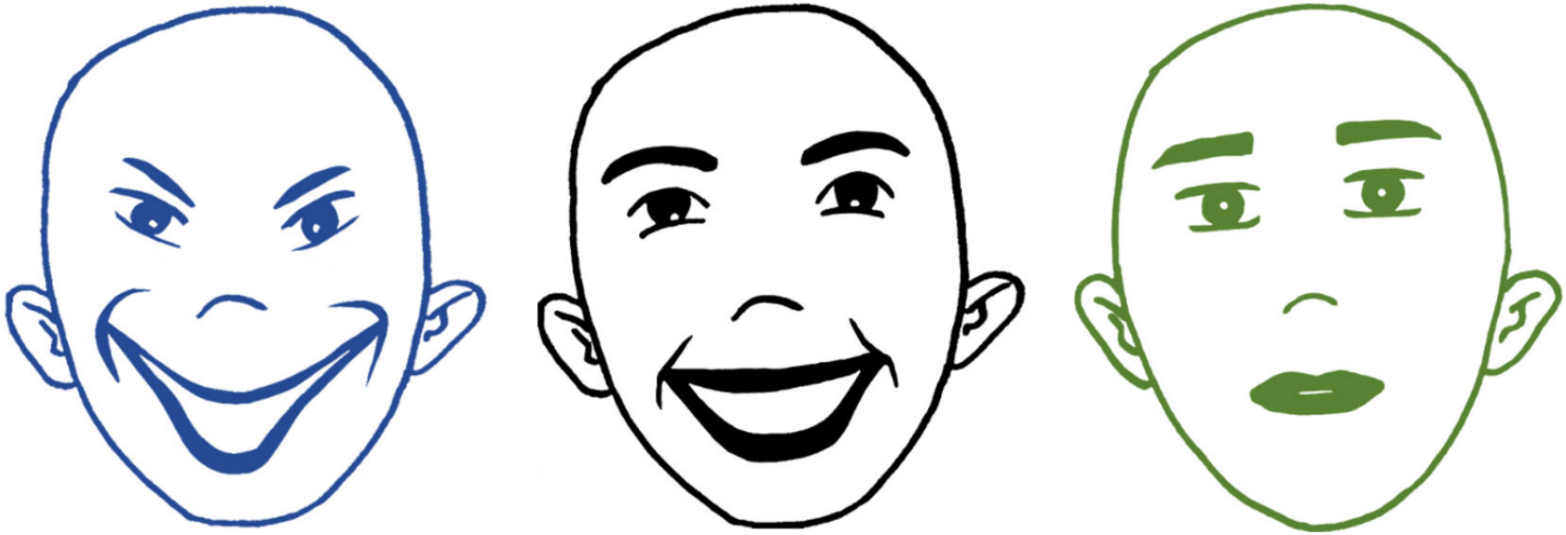
Display of response options for recognition task. *Note.* Blue = mean face; Black = nice/friendly face; Green = neutral face. Note that order and color of faces were randomized between participants. (For interpretation of the references to color in this figure legend, the reader is referred to the web version of this article.)

The Social Intention in Laughter Task (SILT) was adapted from previous research (Plate et al., 2022) for use with young children. The task was administered remotely, with research assistants sharing their computer screen and making selections based on child responses. First, we included an audio check where participants heard a word (“apple”), which the child repeated. The task did not advance unless the child provided the correct response. Then, children were randomly assigned to complete the recognition or social preference condition first. In the recognition task, children were introduced to the response options, which were 3 line-drawn faces (friendly smiling, mocking smiling, and neutral) with outlines in either green, blue, or black (Fig. 1). The order of the faces on the screen and the color assigned to each face were randomized between children (but consistent within participant). The research assistant introduced the faces by saying, *“Let’s check how these people are feeling. Who looks like they are laughing with a nice or friendly face?”* Children could provide a verbal response of the color or point to the face, which their parent then relayed to the research assistant. They were also asked, *“Who looks like they are laughing with a mean or unfriendly face?”* and *“Who looks like they are not laughing and have a calm face?”* If children responded incorrectly, the research assistant provided corrective feedback by stating the color of the correct face (“Nice try! The [color of the face] is laughing with a nice face”). To reinforce the response options (not intended as a manipulation check), the research assistant also said, *“Now let’s say the emotions together! The [color of face] is laughing with a nice face, the [color of face] is laughing with a mean face, and the [color of the face] is not laughing and has a calm face”.* The research assistant then provided instructions for the task*: “In this game you will hear some people laughing. I want you to tell me which face is laughing”.* Children heard each of 6 laughter clips (three of each type; Table 1) and could indicate their response by saying the color of the face or pointing (with response relayed by parent). Participants could also respond *“I don’t know”*, in which case the research assistant would record their response and advance the trial. Participants completed one practice trial with the same laughter clip across participants, without feedback, to gain familiarity with the task. For the social preference condition, children heard 6 more laughter clips in randomized order (i.e., different clips from the recognition condition) and the research assistant provided instructions: *“In this game, you will hear a person laughing. I want you to tell me if you want to play with that person”.* A “thumbs up” and a “thumbs down” image appeared on the screen (side of screen randomized between participants). Children could point at a response option, give a “thumbs up” or “thumbs down” themselves, verbally say *“yes”* or *“no”,* or they could say, *“I don’t know”.* Children completed one practice trial with the same laughter clip across participants, without feedback, to gain familiarity with the task. For both tasks, children had to listen to the entire clip before responding and could not repeat the clip. Research assistants skipped the trial if participants took longer than 10 s to respond (<3 % of trials were skipped or participants indicated *“I don’t know”*).

##### Stimuli.

Laughter clips were based on prior studies where adult participants had rated the extent to which laughter clips, originally acquired from a professional online sound library (soundsnap.com) and included audio clips produced for a variety of media, conveyed affiliation and dominance on two 10-point Likert scales (1 = *not at all*, 10 = *very much*) (Wood et al., 2017). We selected four “friendly” clips previously rated as high on affiliation (*M* = 7.39) and low on dominance (*M* = 3.14), four “mean” clips rated low on affiliation (*M* = 2.63) and high on dominance (*M* = 7.69), and four “ambiguous” clips rated near the midpoint of the scale for affiliation (*M* = 5.41) and dominance *(M* = 5.48). Each type of laugher clip included two female-produced and male-produced clips (one of each was used in the Recognition and Social Preference Tasks). Clips were consistent in length (<3 s), volume, and laughter source (i.e., eliminating clips that had been originally developed for animations) (Plate et al., 2022).

#### Callous-unemotional (CU) traits

We assessed child CU traits with the parent-report version of the Inventory of Callous Unemotional traits (ICU; Frick, 2004). This measure includes 24 items with a four-point response scale (1 = *do not agree at all* to 4 = *strongly agree*). The ICU assesses lack of empathy (e.g., “unconcerned about the feelings of others”), uncaringness (e.g., “always tries best” – reverse scored), and lack of emotionality (e.g., “hides feelings”). Consistent with recommendations (Ray & Frick, 2020), we summed all 24 ICU items (α = 0.82).

#### Conduct problems (CP)

We used parent reports on the 5-item CP scale of the Strengths and Difficulties Questionnaire (SDQ; Goodman, 2001), which assesses aggressive behavior and rule-breaking (e.g., “lies or cheats”). We summed items to create CP scores (α = 0.51), which we included as a covariate in analyses to establish specificity of findings to CU traits rather than overall CP severity.

#### Procedure

Participants were recruited through social media advertisements (Meta) in the wider metropolitan area of [masked], flyers posted in the community, and institutionally maintained databases. Parents and children participated in a 30-minute Zoom call with a research assistant who provided instructions and navigated the child and parent through tasks. Written consent was obtained from the parent and verbal assent from the child. After the visit, parents were emailed a survey link (Qualtrics) to complete the questionnaires. Families were compensated $50 for completing the visit and $20 for completing questionnaires. Study procedures were approved by the Institutional Review Board at the University of Pennsylvania.

#### Analytic strategy

For Aim 1, we examined accuracy on the recognition task overall, as well as accuracy for each laughter type using t-tests (Bonferroni-corrected) testing mean accuracy against chance (i.e., .33 since there were three response options). There was no correct response for ambiguous clips, and they were excluded from recognition condition analyses. Next, we ran a logistic mixed effects model regressing trial accuracy (i.e., correct = 1, incorrect = 0) on laughter type (centered to aid in interpreting effects: dominant = −0.5, affiliative = 0.5) to assess whether participants were more accurate at recognizing friendly or mean laughter. To test age differences, we included mean-centered participant age (months) in an interaction term with laughter type. For the social preference task, we included ambiguous clips because the response was subjective, first testing preference to play against chance (chance was .50 because there were two responses, yes or no), next using a logistic mixed effects model regressing desire to play (yes = 1, no = 0) and laughter type (friendly, mean, ambiguous), and finally including mean-centered participant age (months) in an interaction term with laughter type. For Aim 2, we examined main effects of CU traits on recognition and social preference. We also tested whether CU traits interacted with laughter type and age. For all models, we included random intercepts for participant, by-participant random slopes for laughter type (friendly and mean for the recognition task and friendly, mean, and ambiguous for the social preference task), and random intercepts for item (i.e., specific laughter clip). We included CP traits as a covariate in all models with CU traits.

We used R 4.0.3 (R Core Team, 2020), the tidyverse (Wickham et al., 2019), lme4 package (Bates et al., 2014), and ggplot2 (Wickham, 2016) packages. The task was in Psychopy (Peirce et al., 2019). We report how we determined our sample size, all data exclusions, and all manipulations and measures. The study was not preregistered.

## Results

### Children accurately recognize affiliative and dominant laugher

**Fig. 2.**
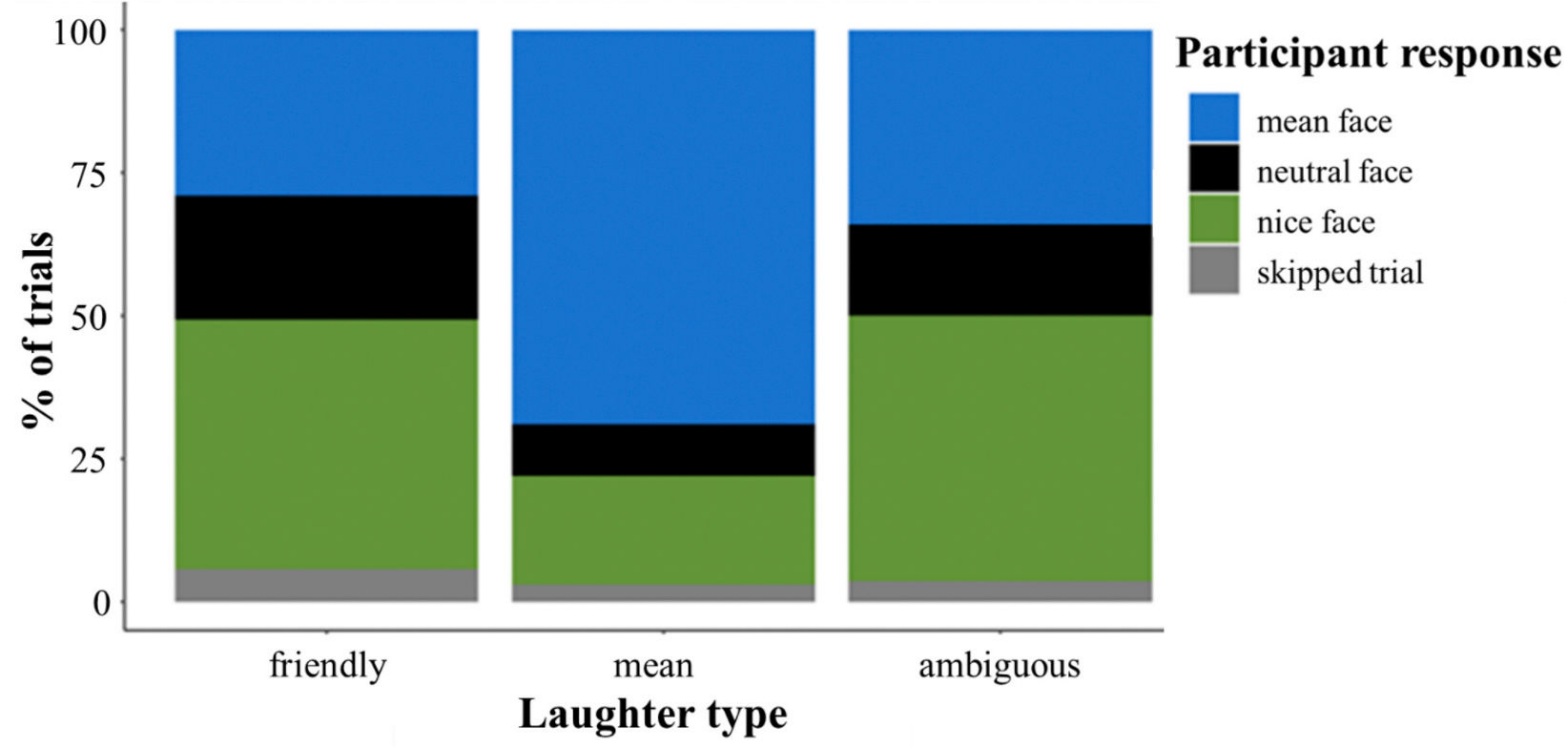
Distribution of participant choices in the recognition task. *Note.* Children had above chance accuracy overall (59 %, *t*(146) = 11.43, *p*_*adj*_ = 0.001, *d* = 1.89) and separately for friendly (46 %, *t*(146) = 4.64, *p*_*adj*_ < 0.001, *d* = 0.77) and mean laughter (71 %, *t*(146) = 12.41, *p*_*adj*_ < 0.001, *d* = 2.03).

**Fig. 3.**
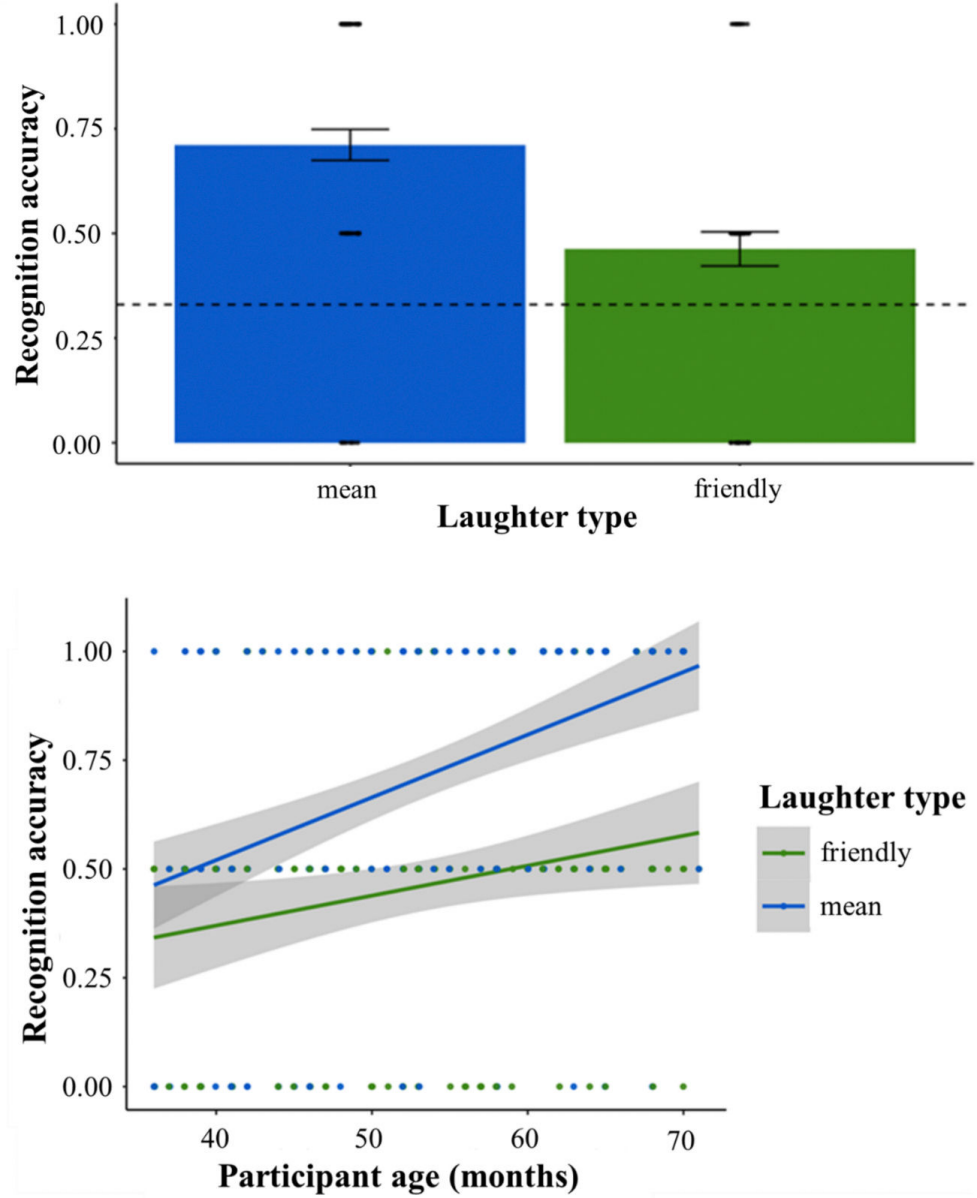
Overall recognition accuracy by laughter type and with increasing age. *Note.* Participants were more accurate recognizing dominant versus affiliative laughter (*OR* = 0.32, *z* = −4.58, *p* < 0.001), and accuracy increased overall with age (*OR* = 1.05, *z* = 5.02, *p* < 0.001). There was an interaction between laughter type and age (*b* = −0.05, *z* = −2.51, *p* = 0.01), such that accuracy gains with age were more pronounced for dominant (*b* = 0.09, *z* = 4.41, *p* < 0.001) versus affiliative laughs (*b* = 0.03, *z* = 2.30, *p* = 0.02).

Consistent with the Aim 1 hypothesis, children recognized different laughter types with greater accuracy than expected by chance overall (59 %, *t*(146) = 11.43, *p*_*adj*_ = 0.001, *d* = 1.89) and for each laughter type separately (friendly: 46 %, *t*(146) = 4.64, *p*_*adj*_ < 0.001, *d* = 0.77; mean: 71 %, *t*(146) = 12.41, *p*_*adj*_ < 0.001, *d* = 2.03; Fig. 2a depicts the choice distributions). Children more accurately recognized mean versus friendly laughter (Table 2) and accuracy increased overall with age (Table 2). There was an interaction between laughter type and age (*b* = −0.05, *z* = −2.51, *p* = 0.01), such that accuracy gains with age were more pronounced for mean (*b* = 0.09, *z* = 4.41, *p* < 0.001) versus friendly laughter (*b* = 0.03, *z* = 2.30, *p* = 0.02) (Fig. 3; Table 3).

### Children prefer to play with affiliative laughers

**Fig. 4.**
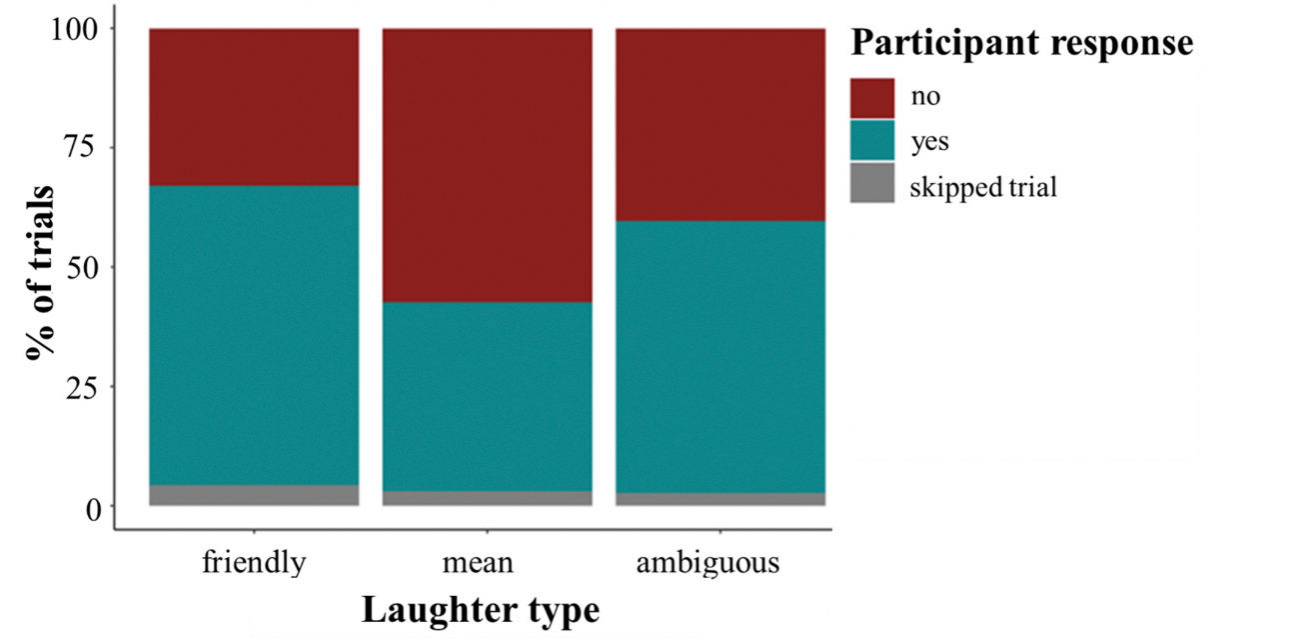
Distribution of participant choices in the social preference condition. *Note.* Children preferred to play with friendly (65 %, *t*(148) = 5.07, *d* = 0.83, *p*_*adj*_ < 0.001) and ambiguous laughers (58 %, *t*(148*)* = 2.67, *d* = 0.44, *p*_*adj*_ = 0.03). Children preferred not to play with mean laughers (41 %, *t*(149) = −2.77, *d* = −0.45, *p*_*adj*_ = 0.03).

**Fig. 5.**
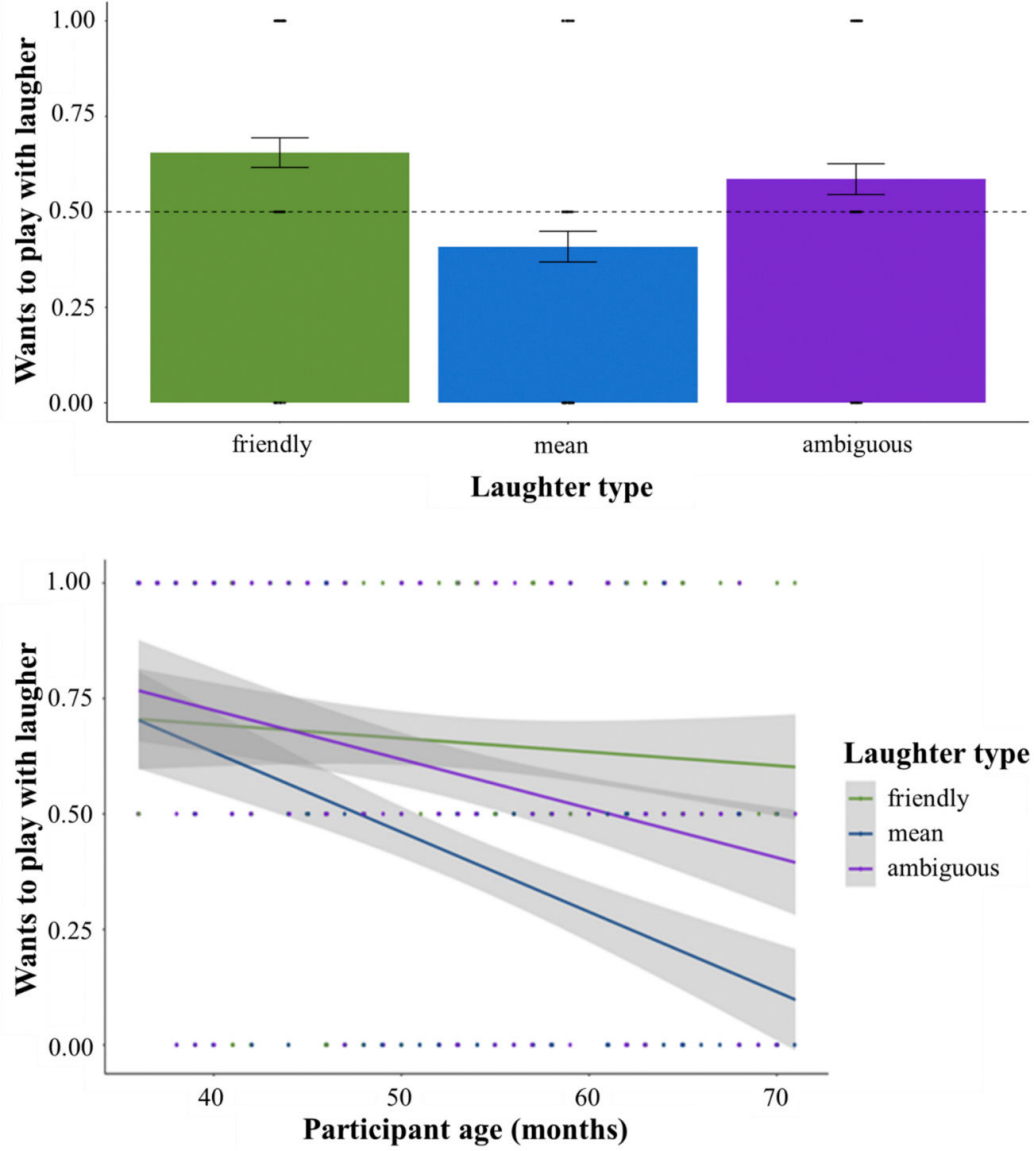
Social preference by laughter type and with increasing age. *Note.* Children preferred to play with friendly (65 %, *t*(148) = 5.07, *d* = 0.83, *p*_*adj*_ < 0.001) and ambiguous laughers (58 %, *t*(148*)* = 2.67, *d* = 0.44, *p*_*adj*_ = 0.03). Children preferred not to play with mean laughers (41 %, *t*(149) = −2.77, *d* = −0.45, *p*_*adj*_ = 0.03). There was a steeper reduction in wanting to play with mean laughers for older children (vs. friendly: *b* = −1.25, *z* = −6.27, *p* < 0.001; vs. ambiguous: *b* = −0.91, *z* = −4.72, *p* < 0.001; friendly vs. ambiguous: *b* = 0.33, *z* = 1.70, *p* = 0.21). Simple slopes indicate that the desire to play with laughers decreases with age for mean (*b* = −0.08, *z* = −5.14, *p* < 0.001) and ambiguous (*b* = −0.05, *z* = −3.54, *p* < 0.001) laughs, but not for friendly laughs (*b* = −0.01, *z* = −0.99, *p* = 0.32).

Also consistent with our Aim 1 hypothesis, children showed significant social preferences to play with friendly (65 %, *t*(148) = 5.07, *p*_*adj*_ < 0.001, *d* = 0.83) and ambiguous laughers (58 %, *t*(148*)* = 2.67, *p*_*adj*_ = 0.03, *d* = 0.44; Fig. 4). Children also preferred not to play with mean laughers (41 %, *t*(149) = −2.77, *p*_*adj*_ = 0.03, *d* = −0.45) (Fig. 5). Overall, with increasing age, children expressed lower preference to play with all laughers (*X*^*2*^*(1)* = 20.41, *p* <0.001), but this effect was driven by a steeper reduction in wanting to play with mean laughers among older children (vs. friendly: *b* = −1.25, *z* = −6.27, *p* < 0.001; vs. ambiguous: *b* = −0.91, *z* = −4.72, *p* < 0.001; mean vs. ambiguous: *b* = 0.33, *z* = 1.70, *p* = 0.21, Fig. 5; Table 4).

### CU traits moderate laughter recognition but not social preferences

**Fig. 6.**
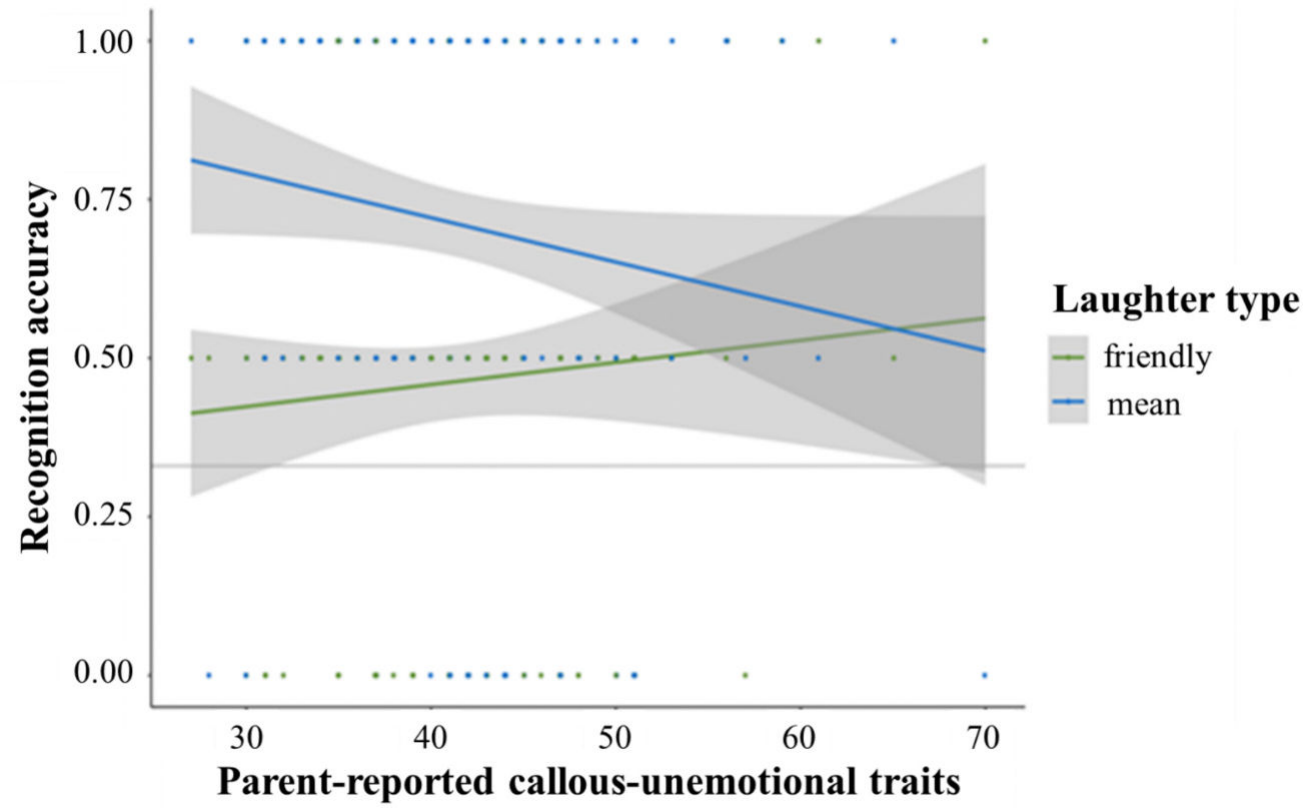
Interaction between laughter type and CU traits on recognition accuracy. *Note.* Higher levels of parent-reported CU traits were related to lower recognition accuracy for mean laughter (*b* = −0.29, *z* = −2.03, *p* = 0.04). CU traits were not associated with recognition accuracy of friendly laughter (*b* = −0.05, *z* = −0.45, *p* = 0.65). Gray line indicates chance (0.33).

Overall, CU traits were unrelated to recognition accuracy for laughter (*b* = −0.13, *z* = −1.78, *p* = 0.08; Table 5). However, the interaction between laughter type and CU traits was just below the threshold for statistical significance (omnibus: *b* = 0.25, *z* = 1.94, *p* = 0.05), such that increasing levels of CU traits were related to poorer recognition of mean laughter (*b* = −0.29, *z* = −2.03, *p* = 0.04) but not friendly laughter (*b* = −0.05, *z* = −0.45, *p* = 0.65) (Fig. 6; Table 6). Additional probing of the significant interaction revealed that children with low CU traits (<1 *SD* below the mean) showed significantly better recognition of mean versus friendly laughter (*b* = −1.28, *z* = −2.58, *p* = 0.01), while children with high CU traits (>1 *SD* above the mean) showed no difference in accuracy (*b* = −0.53, *z* = −1.03, *p* = 0.31). CU traits did not interact with age in relation to recognition accuracy (*b* = 0.00, *z* = 0.04, *p* = 0.97; the three-way interaction between CU traits, age, and laughter type was also not significant, *b* = −0.02, *z* = −1.32, *p* = 0.19). For the social preference task, there was no main effect of CU traits (*b* = −0.01, *z* = −0.13, *p* = 0.89) and no significant interaction with laughter type (*X*^*2*^(2) = 1.44, *p* = 0.49) and age (*b* = −0.02, *z* = −0.23, *p* = 0.82), and the three-way interaction was not significant (*X*^*2*^(2) = 2.47, *p* = 0.29).

## Discussion

We examined laughter recognition and social preferences of 3- to 5-year-old children during a new laughter categorization task, as well as differences in responding as a function of CU traits. First, young children showed above-chance accuracy in recognizing friendly and mean laughter. This finding is consistent with a large prior literature showing that children generally can detect the valence and arousal conveyed by different emotional cues during the preschool period, including from facial and bodily expressions of emotion (Parker et al., 2013; Riddell et al., 2024). Children were more accurate at recognizing mean laughter, which conveys mocking, unfriendliness, and/or social dominance (Plate et al., 2022; Wood et al., 2017; Wood & Niedenthal, 2018). This finding mirrors evidence for a purported negativity bias in attending to and interpreting socioemotional cues, which may represent an evolutionarily adaptive function that allows us to avoid harm within our environment (Vaish et al., 2008). Consistent with developmental patterns of emotion recognition for other types of social and emotional signals (Riddell et al., 2024), children showed increasing accuracy across ages 3–5, particularly for mean laughter.

Second, children exhibited social preferences (i.e., said yes, they wanted to play with the laugher) for friendly and ambiguous laughers, but not mean laughers, with whom they expressed a significant preference *not* to play. This finding is broadly consistent with the role of laughter in promoting social bonding among humans and social nonhuman animals (Dunbar et al., 2021; Dunbar & Mehu, 2008; Dunbar et al., 2012; Davila-Ross & Palagi, 2022). Our results extend prior work by demonstrating children’s reluctance to play with mean laughers, which highlights children’s ability to use different types of laughter as social cues to guide behavior. Indeed, as with recognition of laughter, children may be particularly sensitive to those who might be socially harmful, which may be more salient as children enter preschool and their social networks expand to include “friends and enemies” (Hartup & Abecassis, 2002).

Third, we found no overall association between CU traits and laughter recognition. This finding is consistent with prior work showing that adolescents with high CU traits were as good as typically developing peers at distinguishing genuine versus posed laughter (O’Nions et al., 2017), indicating intact recognition of other laughter features. Additionally, CU traits were not related to children’s social preferences. However, with increasing levels of CU traits, we found that young children showed poorer recognition of mean laughter and significantly less differentiation between mean and friendly laughter compared to children with low CU traits (though the full interaction reached just below the threshold for statistical significance). These findings are in line with evidence that young children high on CU traits show poor recognition of potentially negative reinforcement cues from the social environment, which prior studies have operationalized in the form of facial expressions of sadness (Powell et al., 2023), fear (White et al., 2016), and anger (Bedford et al., 2021), as well as time-out (Kimonis et al., 2024) and teacher discipline (Hwang et al., 2021). Our findings are also consistent with a prior study where adults higher on psychopathic traits (i.e., manipulative, callous, impulsive) had difficulty distinguishing friendly and mean laughter (Plate et al., 2022). More broadly, struggling to differentiate between social cues, including those contained within laughter, likely undermines how children high on CU traits engage with others to develop social relationships.

Strengths of the current study include our task-based measure, tested using a within-subjects experimental design, and our focus on 3- to 5-year-old children whose social skills and emotion recognition capabilities are rapidly improving, making this an ideal population to test laughter recognition and social intentions. However, there are several important study limitations. First, our community sample had relatively low CU traits and CP, as well as a significant proportion of parents with high educational attainment and family income. To increase generalizability, replication of findings is needed among more economically diverse samples, as well as in children with clinically significant CP. Second, all the laughter was produced by adults. Some evidence suggests that children show differential emotion recognition for signals conveyed by same-aged actors (Hoehl et al., 2010). Future studies are needed that can test this hypothesis in relation to laughter recognition. Third, to reduce participant burden and maintain children’s attention through the task, we included only 6 trials for each study condition (i.e., 2 affiliative, 2 dominant, and 2 ambiguous trials in each of the recognition and social preference conditions). Despite evidence that children were able to accurately detect the type of laugher and expressed contingent social intentions towards laughers, future work is needed that includes a greater number of trials. Finally, the laughs were posed laughs and presented on Zoom (it is possible that Zoom settings can affect audio quality). A next step of this research is to better understand laughter recognition and response in naturalistic in-person interactions.

In sum, preschool-aged children showed above-chance recognition of friendly and mean laughter and wanted to play preferentially with friendly and ambiguous laughers, and not with mean laughers. Children with higher CU traits had difficulties distinguishing mean and friendly laughter, which may help to explain some of their early social difficulties. Future studies are needed to replicate our findings and extend this paradigm to include child-produced laughter, add more trials, and recruit more diverse samples.

## Supplementary Material

1

## Figures and Tables

**Table 1:** Average affiliation (friendly) and dominance (mean) ratings of clips based on laughter type.

Recognition Task		
		
Laughter type	Mean affiliation rating	Mean dominance rating

Affiliative	7.28	3.22
Dominant	2.10	7.66
Ambiguous	5.29	5.81
Social preference task		

Laughter type	Mean affiliation rating	Mean dominance rating

Affiliative	7.51	3.05
Dominant	3.16	7.72
Ambiguous	5.53	5.16

*Note.* Laughter clips for both tasks and ratings (10-point Likert scale, 1 = “not at all”, 10 = “very much”) are from a separate adult sample reported by Wood et al. (2017).

**Table 2:** Recognition accuracy is higher for older participants and mean laughter. (Aim 1).

Predictors	Odds ratios	95 % confidence interval	z-value	p-value

(Intercept)	0.1	0.03–0.29	−4.21	<0.001
Laughter type	0.32	0.20–0.52	−4.58	<0.001
Participant age (Months)	1.05	1.03–1.07	5.02	<0.001
Participant sex	1.14	0.78–1.66	0.68	0.496
Task order	1.06	0.73–1.54	0.31	0.755

*Note.* Table shows results from logistic mixed effects model regressing trial accuracy (i.e., correct = 1, incorrect = 0) on laughter type (mean = −0.5, friendly = 0.5), participant age (in months) participant sex (male = −0.5, female = 0.5) and task order (social preference first = −0.5, recognition first = 0.5). There were no main effects of participant sex or task order.

**Table 3:** Accuracy gains with age were more pronounced for mean laughter (Aim 1).

Predictors	Odds ratios	95 % Confidence Interval	z-value	p-value

(Intercept)	0.09	0.03–0.27	−4.39	<0.001
Laughter type	4.01	0.52–30.74	1.34	0.181
Participant age (Months)	1.05	1.03–1.08	5.21	<0.001
Laughter type*participant age	0.95	0.92–0.99	−2.51	0.012

*Note.* Table shows results from logistic mixed effects model regressing trial accuracy (i.e., correct = 1, incorrect = 0) on laughter type (mean = −0.5, friendly = 0.5), participant age (in months), and the interaction between laughter type and participant age.

**Table 4:** Play preferences were affected by laughter type, participant age, and their interaction (Aim 1).

Predictors	X^2^	df	z-value

Laughter type	40.271	2	<0.001
Participant age (months)	20.417	1	<0.001
Laughter type*participant age	12.763	2	0.002

*Note.* Table shows results from logistic mixed effects model regressing desire to play (i.e., yes = 1, no = 0) on laughter type (mean, friendly, ambiguous), participant age (in months) participant sex (male = −0.5, female = 0.5), and the interaction between laughter type and participant age.

**Table 5:** CU traits were not related to recognition accuracy overall (Aim 2).

Predictors	Odds ratios	95 % confidence interval	z-value	p-value

(Intercept)	0.15	0.04–0.60	−2.69	0.007
CU Traits	0.88	0.76–1.01	−1.78	0.076
Conduct Problems	2.21	0.98–5.02	1.9	0.057
Participant Age (Months)	1.05	1.03–1.06	4.93	<0.001

*Note.* Table shows results from logistic mixed effects model regressing trial accuracy (i.e., correct = 1, incorrect = 0) on CU traits, conduct problems, and participant age (in months).

**Table 6:** Higher CU traits were related to poorer recognition of mean laughter (Aim 2).

Predictors	Odds ratios	95 % confidence interval	z-value	p-value

(Intercept)	0.14	0.03–0.60	−2.63	0.009
Laughter type	0.04	0.00–0.34	−2.96	0.003
CU traits	0.86	0.73–1.01	−1.87	0.061
Conduct problems	2.33	0.96–5.64	1.87	0.061
Participant age (months)	1.05	1.03–1.07	4.97	<0.001
Laughter type*CU traits	1.28	1.00–1.65	1.94	0.052

*Note.* Table shows results from logistic mixed effects model regressing trial accuracy (i.e., correct = 1, incorrect = 0) on laughter type (mean = −0.5, friendly = 0.5), CU traits, conduct problems, participant age (in months), and the interaction between laughter type and CU traits.

## Data Availability

De-identified datasets and analysis scripts are on Open Science Framework (https://osf.io/f9kpr/overview). The experimental task will be shared upon request.

## Footnotes

1 Full sample information: child age in months, *M*=53.02, *SD*=9.90, *range*=36–71; female, *n*=81, 50.0%; Asian, *n*=10, 6.2%; Black, *n*=20, 12.3%; White, *n*=108, 66.7%; Biracial or more than one race, *n*=24, 14.8%). Parents were biological mothers (*n*=159, 98%) or fathers (*n*=3, 2%) who reported being from the following racial groups: Asian (*n*=23; 14.2%), Black (*n*=17; 10.5%), White (*n*=115; 71.0%), and biracial or more than one race (*n*=7, 4.3%). More than half of parents (67%) reported having a graduate-level degree, 22% had a Bachelor-level degree, 4% had completed some college, 3% had an Associate’s degree or technical training, 4% had a high school degree or less, and 1% declined to report. Average monthly household income was $14,224 (*SD*=$9,203).

## Appendix A. Supplementary data

Supplementary data to this article can be found online at https://doi.org/10.1016/j.jecp.2025.106395.
